# Efficacy of hyperbaric oxygen therapy combine with negative pressure wound therapy in chronic wound: A randomized controlled trial

**DOI:** 10.1016/j.jpra.2025.05.001

**Published:** 2025-05-08

**Authors:** Thanaporn Riansrithongkham, Pangrum Yongchareon, Ajana Sivadechathep, Anucha Likitvong, Terasak Mahamongkol, Chatchai Pruksapong

**Affiliations:** a Division of Plastic and Reconstructive Surgery, Department of Surgery, Phramongkutklao Hospital and College of Medicine, Bangkok, Thailand; b Maritime Medicine Group, Department of Surgery, Somdech Phra Pinklao Hospital, Bangkok, Thailand; c Hyperbaric Oxygen Medicine Center, Department of Surgery, Somdech Phra Nangchao Sirikit Hospital, Chonburi, Thailand

**Keywords:** Hyperbaric oxygen therapy, Negative pressure wound therapy

## Abstract

**Background:**

Chronic wounds present a significant challenge, resulting in prolonged care, extended hospital stays, and substantial financial and psychosocial burdens for patients and the healthcare system. Despite various techniques available to accelerate wound healing and reduce hospital stays, no standard approach for chronic wound care has been universally agreed upon. Negative pressure wound therapy (NPWT) is well-known, but its effectiveness as a primary treatment is uncertain. Hyperbaric oxygen therapy (HBOT) is another adjunctive technique that increases oxygen supply to wounds to promote healing, similar to NPWT. This study evaluates the effectiveness of combining HBOT with NPWT in treating chronic wounds.

**Methods:**

This prospective multicenter randomized controlled trial involved patients with chronic wounds requiring NPWT. Participants were randomly assigned to receive either HBOT with NPWT or NPWT alone. The primary objective was to compare wound healing rates, with secondary outcomes including pain scores and bacterial contamination.

**Results:**

Each study group included twenty-four patients. The analysis showed a significantly higher wound healing rate in the NPWT+HBOT group compared to the NPWT-only group (11.81 % vs. 8.54 % on day 3, 15.35 % vs. 11.17 % on day 6, 16.44 % vs. 12.14 % on day 9, 20.15 % vs. 14.9 % on day 12; *P* = 0.001). There were no significant differences in pain scores or bacterial contamination between the groups at any time point.

**Conclusion:**

Combining NPWT and HBOT significantly improves the rate of wound healing compared to NPWT alone in chronic wounds, with no significant differences in pain or infection rates between the two treatment groups.

## Background

Chronic wounds demonstrate a disrupted healing process, failing to achieve a sustained anatomical and functional outcome within the typically accepted timeframe of three months. They are unable to advance through the sequential stages of healing and instead become stuck in a perpetual inflammatory state, proving stubbornly resistant to treatment despite proper wound care. Numerous factors, including chronic illnesses, vascular problems, diabetes,^1^malnutrition, aging, and local issues like pressure, infection, and swelling, can impede the healing process.^2^ The resulting tissue injury prolongs and intensifies the inflammatory stage of the wound, marked by a significant influx of neutrophils along with reactive oxygen species and harmful enzymes, thus perpetuating the cycle.^3^ However, resolving the primary harmful factor can effectively promote healing in many chronic wounds.^4^

Non-healing ulcers pose significant challenges for patients and their families, causing pain, infections, functional impairment, financial strain, and often resulting in amputations or sepsis. These chronic wounds primarily stem from prominent health issues such as an aging population, obesity, and diabetes.^5^ As these health concerns continue to rise globally, so does the prevalence of non-healing pressure, venous, and diabetic ulcers. Regrettably, the importance of chronic wounds is often overshadowed by their underlying causes—their economic burden is poorly documented, and there is a lack of adequate care and education.^6^ Nonetheless, chronic wounds persist as a silent epidemic, profoundly affecting the quality of life for over 40 million people worldwide.^7^

Negative pressure wound therapy (NPWT), also called vacuum‐assisted wound therapy, is a wound‐dressing concept used worldwide that continuously or intermittently applies sub‐atmospheric pressure to the surface of a wound in order to promote healing. Numerous prior studies,^8^^,^^9^ including meta-analyses, have demonstrated the benefits of negative pressure wound therapy in enhancing and expediting wound healing, particularly among patients with chronic wounds.

However, physicians continue to explore techniques to enhance wound healing, aiming to complement the effects of negative pressure wound therapy and decrease failure rates. Hyperbaric oxygen therapy is one such approach, utilized to treat chronic wounds by boosting blood oxygen levels. Previous publications have demonstrated the efficacy of hyperbaric oxygen therapy in wound healing.^10^, ^11^, ^12^

Our hypothesis is that combining both techniques will mutually enhance wound oxygenation. Negative pressure wound therapy offers local effects, while hyperbaric oxygen therapy provides systemic benefits. Therefore, this study aims to evaluate whether the combination of hyperbaric oxygen therapy with negative pressure wound therapy improves wound healing compared to using negative pressure wound therapy alone or not?

## Materials and methods

### Patients and study design

This prospective multicenter randomized controlled trial was conducted at Phramongkutklao Hospital, Somdech Phra Nanchao Sirikit Hospital and Somdech Prha Pinklao Hospital, between June 2021 and March 2024. Institutional review board approval: The study protocol was registered in the Thai Clinical Trials Registry (TCTR20240411002). All participants provided written informed consent to take part in this study and for the publication of the photographs.

For the selection of participants for this study, the following inclusion criteria were applied: age 20 to 80 years, developed chronic wound. The exclusion criteria included the following: Had previous surgery in study wound area, immunocompromised host, underlying bleeding disorder, or currently using anti-platelet or anti-coagulant therapy.

Sample sizes were calculated prior to enrolling study participants using reference data from previous literature^13^ and using the clinical superiority design formula using G*power program (Erdfelder, Faul, & Buchner,1996) and applying a sample size calculation formula,^14^^,^^15^ resulting in the need for 24 participants in each study group.

### Randomization

After enrolling participants in the study, they were divided into two groups. The control group received negative pressure wound therapy alone, while the study group received both negative pressures wound therapy and hyperbaric oxygen therapy. We utilized stratified block randomization, with the randomization sequence generated using computer software and stratified according to our center office at Phramongkutklao Hospital. The blocks varied between 4 and 6 to prevent prediction. Sequentially numbered, opaque, sealed envelopes (SNOSE) were employed. A research coordinator at each study site was contacted to request randomization when patients were ready to begin treatment.

### Study protocol and blinding

At the beginning of treatment, demographic information and standard blood chemistry of participants were recorded. To maintain blinding, outcome assessors and data analysts were unaware of the treatment group assignments. Independent outcome assessors, who was not involved with the patients or the study, evaluated the primary outcome, wound size, on day 0 and subsequently every 3 days in both study groups (recorded as day 3, day 6, day 9, day 12, and day 15), completing the assessment two weeks after treatment initiation. The percentage of wound healing was calculated and recorded at each assessment time point. Secondary outcomes included pain scores, which were also recorded, and bacterial wound cultures, which were performed each time the vacuum dressing was changed.

### Interventions and measurement

**Figure 1:**
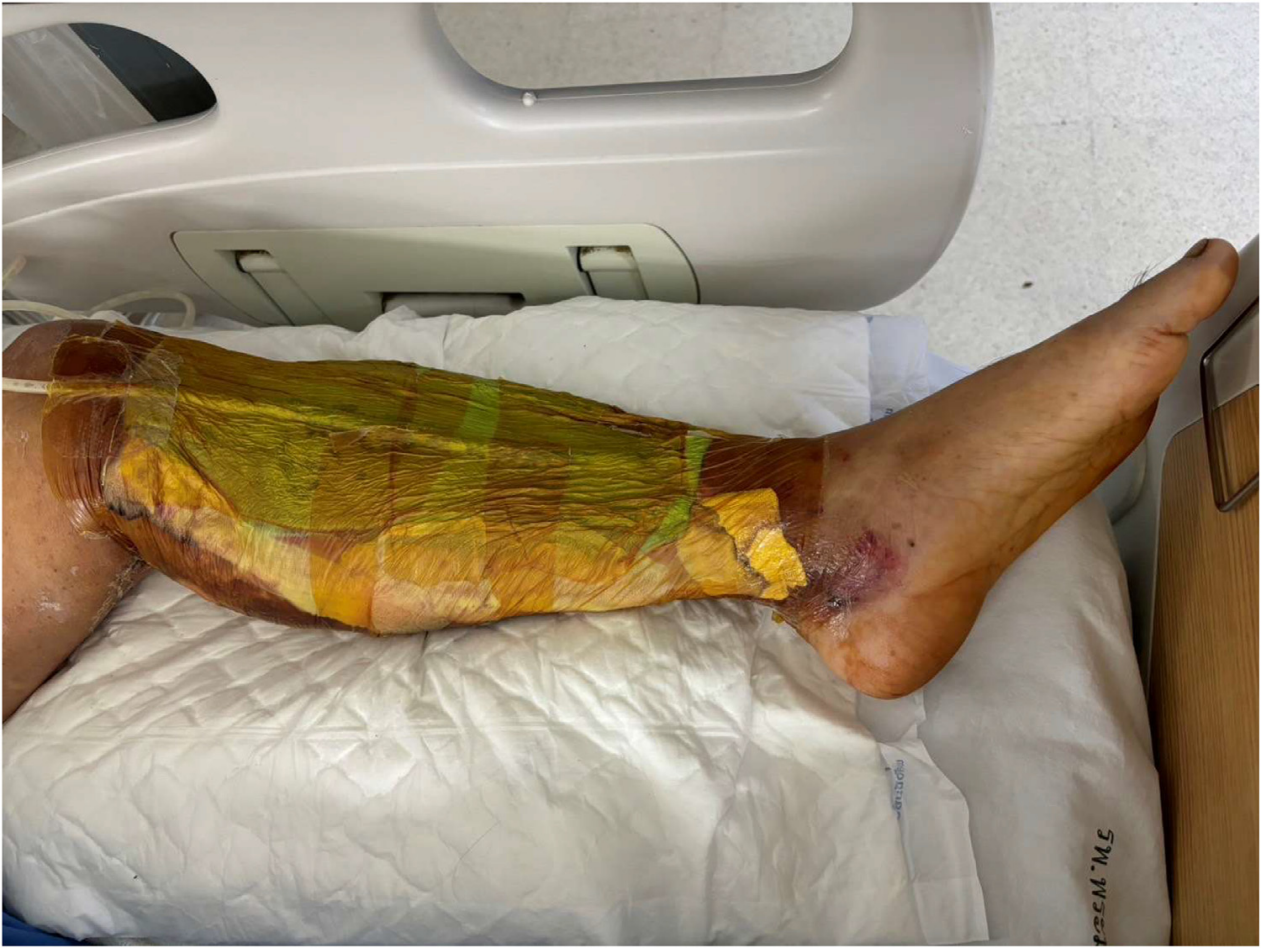
Negative pressure wound dressing.

**Figure 2:**
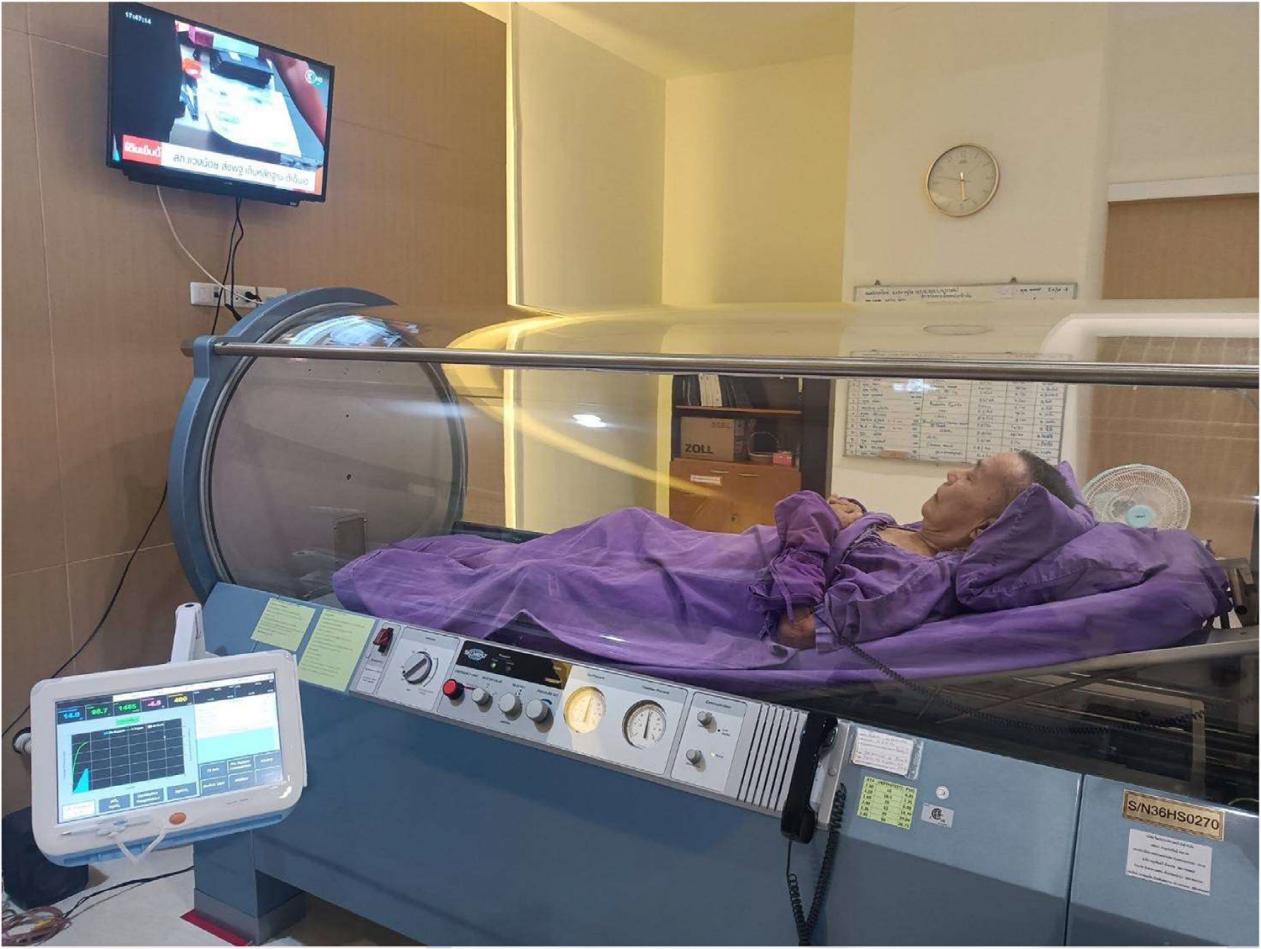
Oxygen chamber in Hyperbaric oxygen therapy.

**Figure 3:**
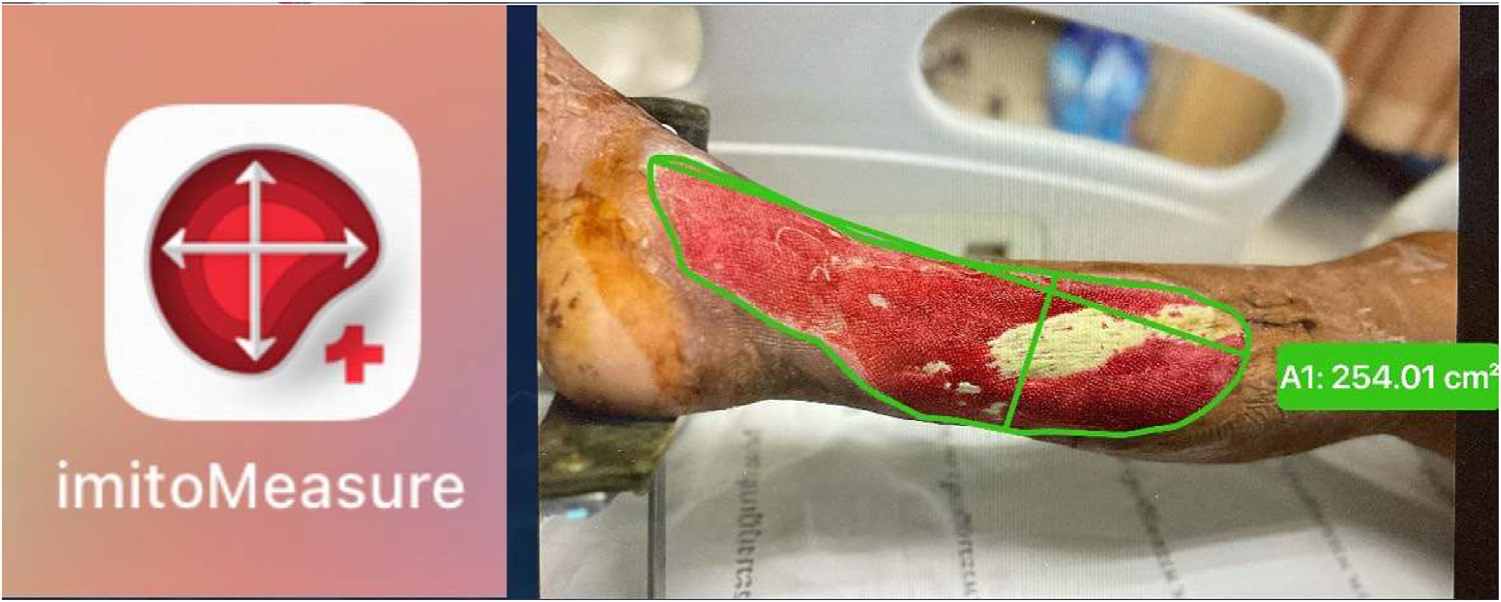
Measurement method of wound size.

In the control group [NWPT], patients underwent dressing with negative pressure wound therapy utilizing a continuous wall-type vacuum suction dressing. The procedure involved placing foam directly on the wound, covering and sealing it with an adhesive film (Figure 1). A drainage tube connected to a portable vacuum pump, set at approximately 125 mmHg, facilitated the removal of air pressure and wound fluids. Suction was applied for 24 hours per day, allowing patients time for showering, and using the toilet. In the study group [NWPT+HBOT], alongside negative pressure wound therapy following the same protocol as the control group, patients underwent hyperbaric oxygen therapy. They received 90 min of 100 % oxygen at a pressure equivalent to being under 33 feet of seawater (2.0 ATA). Oxygen was administered in 30-minute intervals with 10-minute air brakes in between. Treatment occurred from Monday through Friday using Monoplace chamber: model 3600HR serial 36HS0270 (Figure 2). The primary objective outcome, wound area (wound size), was measured using imitoMeasure (Imito AG Corp., Geneva, Switzerland) (Figure 3). which is an application that uses a picture of the wound along with a reference point, such as a ruler, to measure its dimensions. The application calculates the length, width, and circumference of the wound based on the scale provided by the ruler. In both treatment groups, before enrollment, participants’ wounds were debrided to remove all necrotic tissue. Participants received Augmentin as a prophylactic intravenous antibiotic. For those allergic to Augmentin, Clindamycin was used instead.

### Statistical analysis

The baseline characteristics of the patients were described in terms of mean values for age and body mass index (BMI), and the sizes of individual wound areas were compared. Categorical data were assessed using the chi-square test or Fisher’s exact test, as appropriate. Continuous data were analyzed using either the *t*-test or the Wilcoxon rank-sum test, depending on the distribution of the data. Statistical analysis was conducted using IBM SPSS Advanced Statistical Software Version 23.0 (IBM Corp., Armonk, N.Y.).

## Results

**Figure 4:**
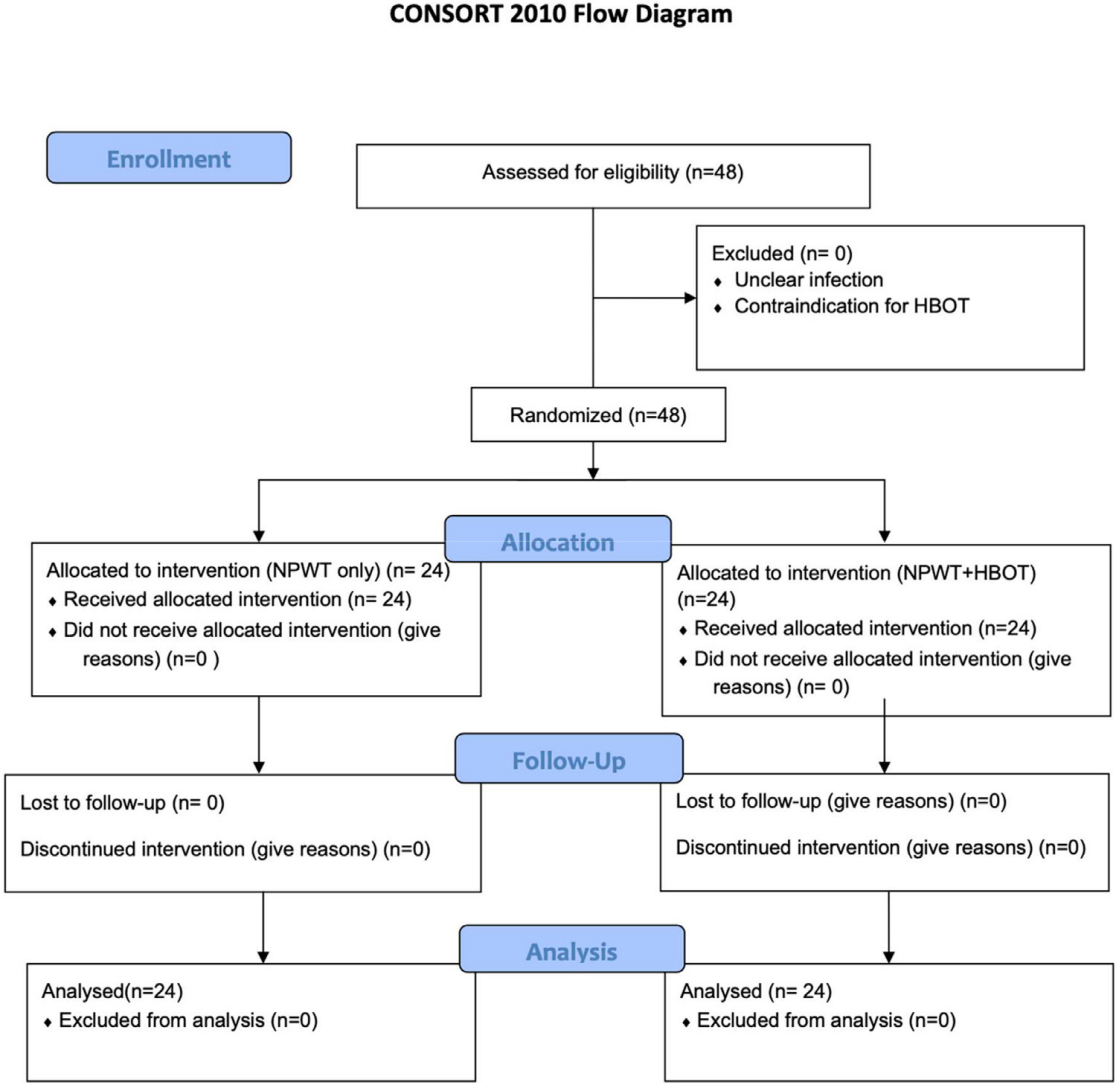
Consolidated standards of reporting trials flow diagram.

Forty-eight patients were enrolled in the study, with twenty-four patients randomized into each treatment group (NPWT+HBOT vs. NPWT) (Figure 4). The average age of patients was 59.58 ± 13.96 years in the NPWT group and 58.25 ± 12.36 years in the NPWT+HBOT group. There were ten female and fourteen male patients in each group. The average BMI was 24.66 ± 2.92 kg in the NPWT group and 25.73 ± 6.49 kg in the NPWT+HBOT group. There were no significant differences in demographic data between the two groups (Table 1).

**Table 1:** Demographic data.

	NPWT (*n* = 24)	NPWT+HBOT (*n* = 24)	*P*-value
sex, *n* (%)			
female	10 (41.67)	10 (58.33)	1
male	14 (58.33)	14 (41.67)	1
age (y), Mean ± SD	59.58 ± 13.96	58.25 ± 12.36	0.083
weight (kg), Mean ± SD	65.52 ± 8.13	65.86 ± 7.83	0.735
height (cm), Mean ± SD	163.92 ± 11.96	160.33 ± 9.33	0.421
BMI, Mean ± SD	24.66 ± 2.92	25.73 ± 6.49	0.323
cause, *n* (%)			
Necrotizing fasciitis	18 (75)	18 (75)	1
Arterial disease	2 (8.33)	2 (8.33)	1
trauma	4 (16.67)	4 (16.67)	1
Comorbid, *n* (%)			
alcohol	6 (25)	4 (16.67)	0.76
smoking	4 (16.67)	6 (25)	0.76
dyslipidemia	8 (33.33)	10 (41.67)	0.86
hypertension	10 (41.67)	10 (41.67)	1
Diabetes mellitus	7 (29.16)	6 (25)	0.92
Hct, Mean ± SD	34.74 ± 5.43	33.96 ± 3.68	0.635
WBC, Median (IQR)	7,400 (5,850–9,950)	7,800 (6,300–8,200)	0.879
platelet, Median (IQR)	338,000 (293,500–413,000)	363,000 (304,500–390,000)	0.898
albumin, Mean ± SD	3.25 ± 0.62	3.38 ± 0.84	0.664
Oxygen saturation at ward (%)	97.4 ± 1.3	97.5 ± 1.4	0.78
Oxygen saturation in chamber (%)	N/A	100	N/A

**Figure 5:**
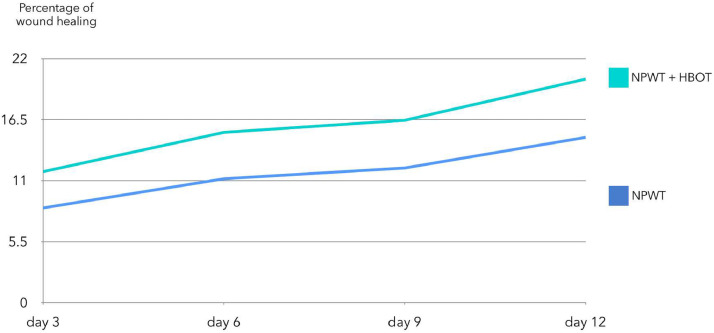
Percentage of wound healing.

**Figure 6:**
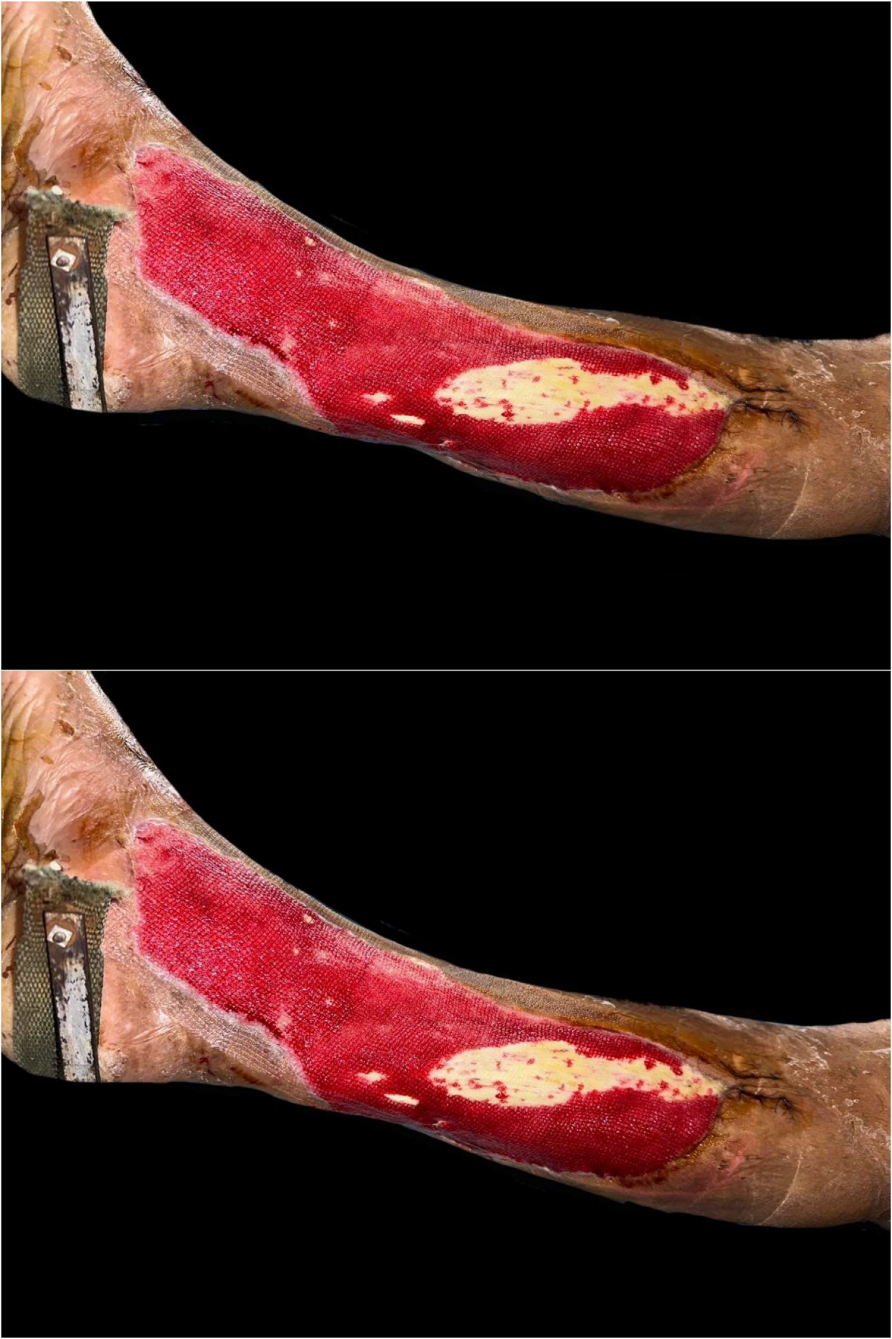

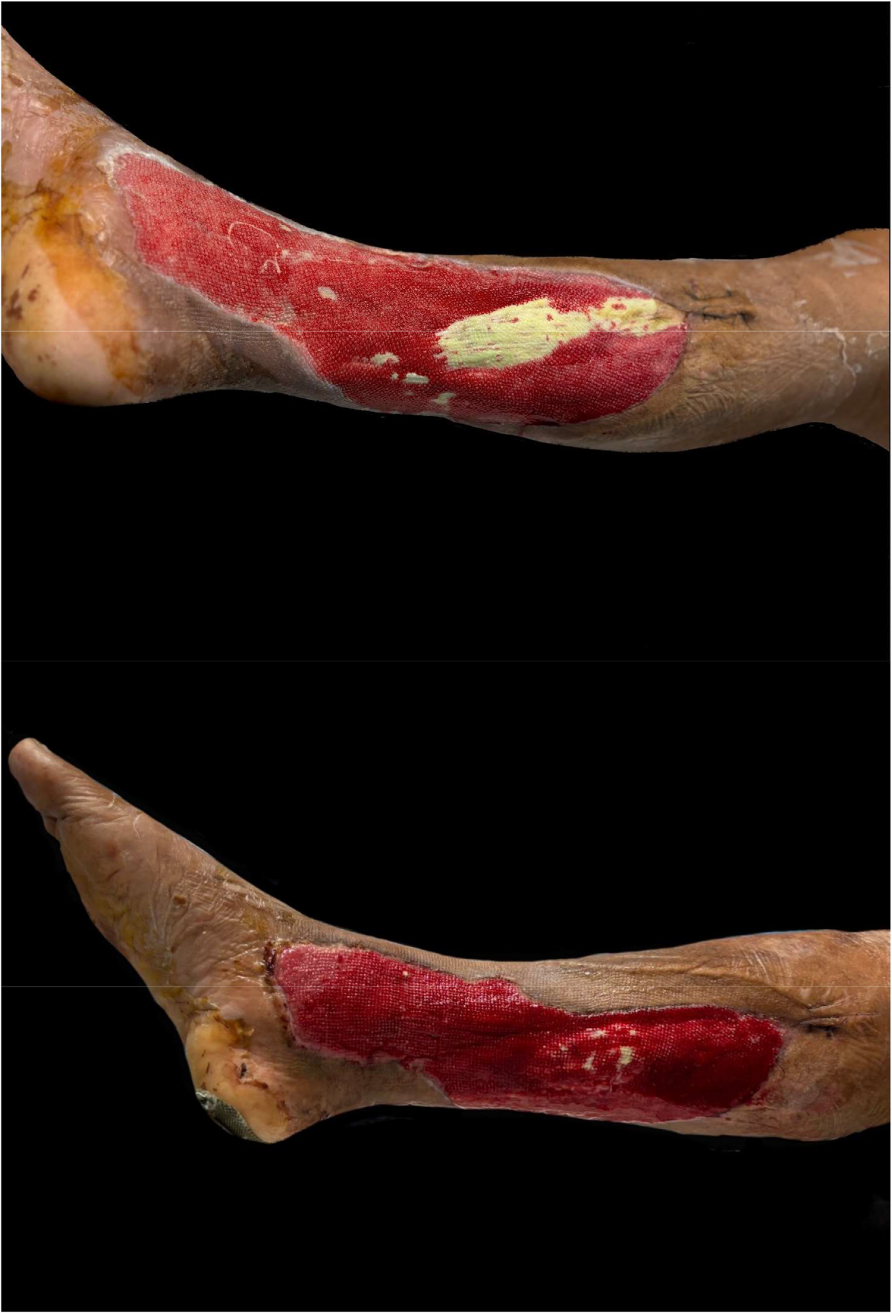

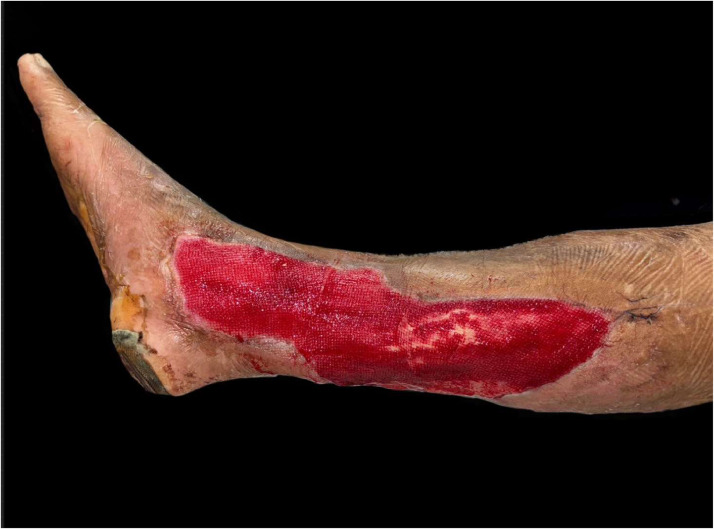
Patient with NWPT and HBOT A 64-year-old male. Underlying Diabetes mellitus and Hypertension with necrotizing fasciitis.

**Figure 7:**
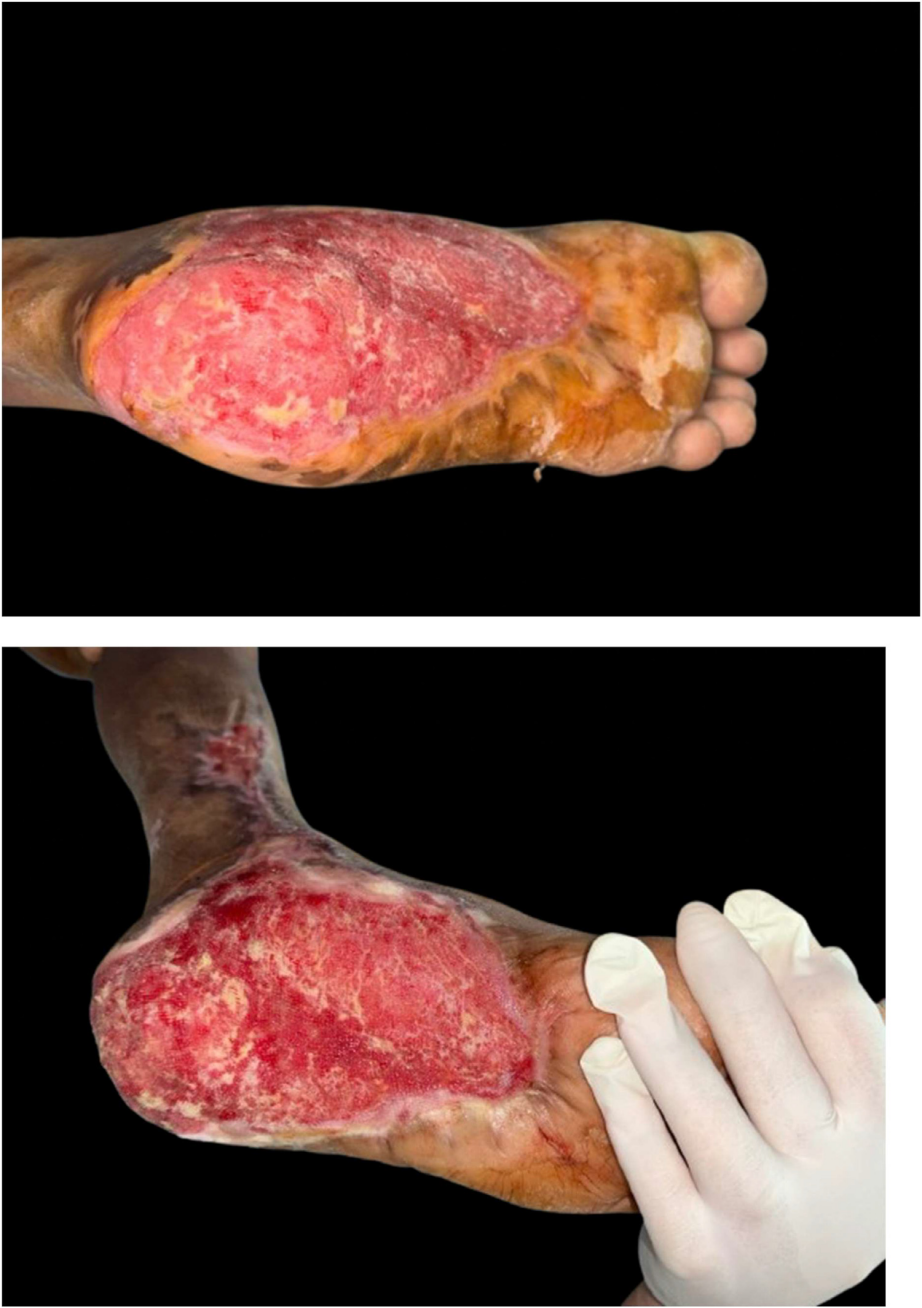

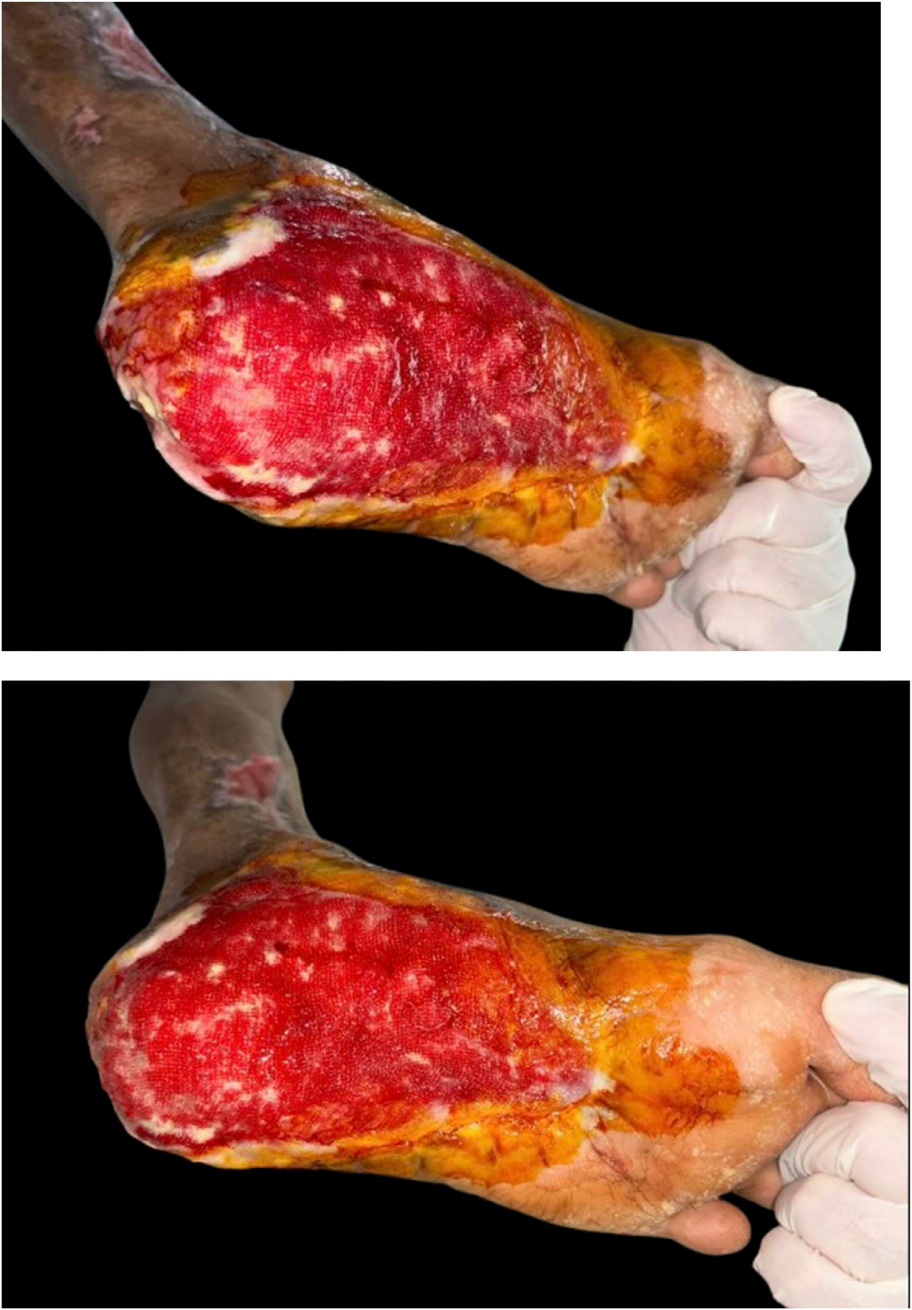

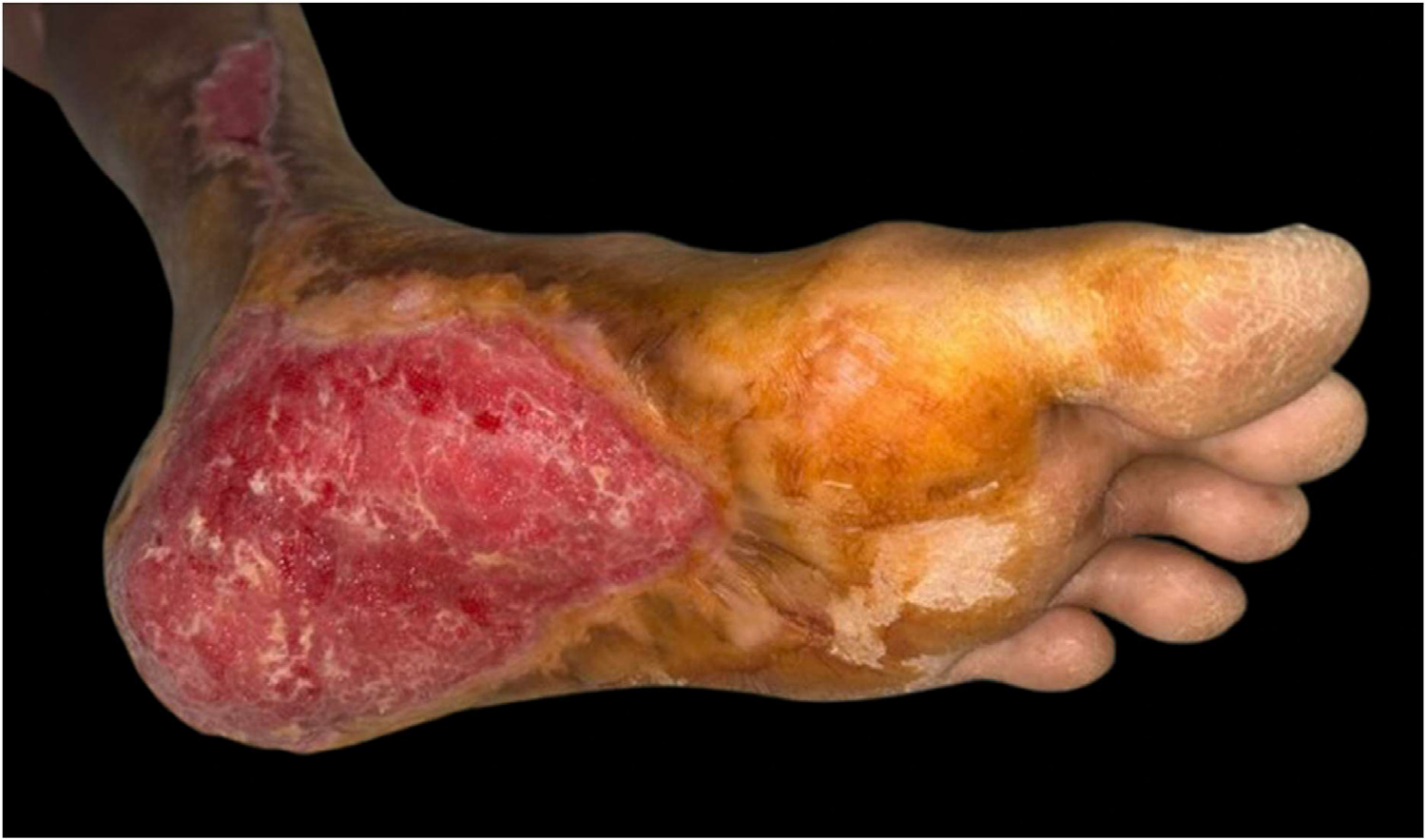
Patients with NWPT and HBOT A 53-year-old female. Underlying Diabetes mellitus.

The percentage of wound healing was significantly higher in the NPWT+HBOT group from day 3 of treatment onwards. Specifically, it was 8.54 % (4.4–13.65) in the NPWT group and 11.81 % (8.98–14.02) in the NPWT+HBOT group on day 3 (*P* value < 0.001), 11.17 % (6.44–15.47) in the NPWT group and 15.35 % (9.32–19.51) in the NPWT+HBOT group on day 6 (*P* value < 0.001), 12.14 % (6.2–20.27) in the NPWT group and 16.44 % (15.56–22.57) in the NPWT+HBOT group on day 9 (*P* value < 0.001), and 14.9 % (7.81–24.67) in the NPWT group and 20.15 % (18.29–24.16)in the NPWT+HBOT group on day 12 (*P* value < 0.001) (Table 2, Figure 5). (Figure 6, Figure 7).

**Table 2:** Percentage of wound healing.

	NPWT		NPWT+HBOT		
	n	Median (IQR)	n	Median (IQR)	*P*-value
%wound healing day 3	24	8.54 (4.2–13.65)	24	11.81 (8.98–14.02)	<0.001
%wound healing day 6	24	11.17 (6.44–15.47)	24	15.35 (9.32–19.51)	<0.001
%wound healing day 9	24	12.14 (6.2–20.27)	24	16.44 (15.56–22.57)	<0.001
%wound healing day 12	22	14.9 (7.81–24.67)	22	20.15 (18.29–24.16)	<0.001

At the start of the study, the pain score was 4 (range: 3.7–5) in the NPWT+HBOT group and 3 (range: 0–5) in the VAC group. By day 3, the pain score decreased to 2.5 (range: 1–5) in the NPWT+HBOT group and 1 (range: 0–3) in the NPWT group. On day 6, the pain score further decreased to 0.5 (range: 0–3) in the NPWT+HBOT group and remained at 0.5 (range: 0–1) in the NPWT group. By day 9, the pain score was 0 (range: 0–3) in the NPWT+HBOT group and 0 (range: 0–1) in the NPWT group. There were no significant differences in pain scores between the beginning and day 9, and no pain was reported after day 9 in either group. (Table 3).

**Table 3:** Pain score.

Pain score	NPWT		NPWT+HBOT		*P*-value
	*n*	Median (IQR)	*n*	Median (IQR)	
Day 0	24	3 (0–5)	24	4 (3–7.5)	0.135
Day 3	24	1 (0–3)	24	1.5^1^^,–^^5^	0.239
Day 6	24	0.5 (0–1)	24	0.5 (0–3)	0.780
Day 9	24	0 (0–1)	24	0 (0–3)	0.745
Day 12	22	0 (0–1)	22	0 (0–3)	0.629

Bacterial cultures were positive in 22 out of 24 cases in each group at the start of treatment. After completing treatment (2 weeks), bacterial cultures remained positive in 5 cases in the NPWT+HBOT group and 6 cases in the NPWT group, with no significant difference between the groups (Table 4).

**Table 4:** Bacterial culture.

	NPWT (*n* = 24) (%)	NPWT+ HBOT (*n* = 24) (%)	*p*-value
Pre-treatment culture	22 (91.67)	22 (91.67)	1.000
Post-treatment culture	6 (25)	5 (20.83)	0.92

## Discussion

Chronic wounds pose life-threatening risks as they often fail to heal within a reasonable timeframe.^7^^,^^16^ Examples of chronic wounds include burns, pressure ulcers, infections, leg ulcers, and more. The management of these wounds is financially burdensome. Annually, the United States spends around USD 20 billion on chronic wound management, while the United Kingdom expends approximately GBP 184 million per year on similar treatments.^6^^,^^17^^,^^18^

Not only is there a life-threatening issue, but there's also an economic problem at play. It affects both personal and family finances directly and indirectly. Directly, there's the cost of treatment, exacerbated by expensive wound dressing products flooding the market without clear evidence of their efficacy in accelerating wound healing. Indirectly, there's the opportunity loss as patients and their families invest time in treatment and rehabilitation, often lasting over a month. This leads to income loss, job insecurity, and even mental health issues like depression and suicidal tendencies. Chronic wounds pose not just individual but national economic burdens, demanding significant budget allocations worldwide. Any method or tool capable of accelerating wound healing would yield global benefits, alleviating the economic strain on individuals and countries alike.

Since its inception in 1997, Negative Pressure Wound Therapy (NPWT), also known as vacuum wound dressing, has undergone significant development. Morykwas and Argenta^19^ introduced the VAC based on a porcine model study. Over the past two decades, numerous clinical and experimental studies have evaluated this technology, demonstrating its efficacy in promoting the healing of acute and chronic wounds.^20^ NPWT works by pulling wound edges together, reducing wound size, facilitating granulation tissue formation, enhancing microcirculation, reducing edema, and removing infectious tissue.^21^

A meta-analysis conducted by Yingchao Yin et al.^22^ revealed the efficacy of NPWT in split-thickness skin grafts. Compared to conventional therapy, NPWT significantly improves the rate of graft take and reduces the need for reoperation when used to cover the wound bed with split-thickness skin graft. The latest systematic review and meta-analysis conducted by Rongli Zhang et al.^23^ confirmed that NPWT significantly reduces patient mortality rates.

One of the principles behind how NPWT assists patients with chronic wounds is by increasing wound oxygenation.^24^^,^ ^25^ Granulation formation relies heavily on oxygenation.^26^ However, the author believes that solely relying on local oxygenation may not be sufficient to maximize and accelerate wound healing. Introducing systemic oxygenation through alternative methods could potentially enhance wound oxygenation. Hence, the consideration of hyperbaric oxygen therapy arises to further boost oxygen levels and aid in improving wound healing.

Hyperbaric oxygen therapy (HBO) is employed to counter hypoxia by administering 100 % oxygen at pressures higher than normal atmospheric levels.^27^ This treatment method augments the amount of dissolved oxygen in the plasma, leading to increased delivery of oxygen to tissues independently of haemoglobin levels.^28^ During HBO exposure, the oxygen pressure in chronic wound tissue significantly rises, mirroring the levels found in normal tissue.^29^ Clinically, this elevation in wound oxygen pressure has been observed to persist for approximately 30 min following HBO therapy. HBO therapy is now widely accepted and routinely utilized to address various conditions associated with ischemia and/or hypoxia.^30^

In a randomized trial conducted by N.A.R. Nik Hisamuddin et al.^13^ on chronic diabetic wounds, the results indicated that Hyperbaric Oxygen Therapy (HBOT) had a significant impact on the healing rate of diabetic foot ulcers. Specifically, there was notable reduction in wound size when compared to administering conventional wound care alone.

At the outset of our study, our principle focused on enhancing both local and systemic oxygenation effects on chronic wounds. Following an extensive review, we found no studies combining the effects of local wound oxygenation through NPWT with systemic oxygenation through HBO therapy. Therefore, we initiated a randomized controlled trial to compare the efficacy of the combined NPWT and HBO procedure against NPWT alone, which solely targets local oxygenation. Due to the nature of the procedure, blinding our patients was not feasible. Thus, we opted for single blinding, with the assessors and observers remaining unaware of the treatment allocations. We conducted a multicentre trial with the aim of ensuring the reliability of our results and their applicability across multiple centres, rather than being limited to a single area or healthcare provider team.

Our results indicate that the combined procedure yields superior outcomes in our primary objective, which is the rate of wound healing, evidenced by a notable reduction in wound size. This reduction in wound size and promotion of granulation tissue formation was evident as early as the initial observation on Day 3. Following the treatment, all patients underwent skin grafting approximately two weeks later. This expedited process facilitated by the combined procedure allowed for a quicker initiation of skin grafting, thereby reducing hospital stays and enabling patients to commence their rehabilitation programs sooner. Consequently, patients could return to work earlier than anticipated.

Regarding our secondary outcome, there was no significant difference observed in pain scores between the two treatment groups. Additionally, the reduction in bacteria culture was equally notable in both groups.

## Limitation

A limitation of our study is that the Hyperbaric Oxygen Therapy (HBOT) provided at our hospital is only available on weekdays. Consequently, we were unable to assess the full-week effect of HBOT. However, all enrolled cases commenced treatment on Mondays, ensuring that patients received HBOT for the initial five days of therapy. Although we calculated the sample size before enrollment began, the small sample size may be a limitation. Future studies with larger sample sizes would be beneficial for chronic wound care research.

## Conclusion

The combination treatment of NPWT and HBO therapy significantly improves the rate of wound healing compared to using NPWT alone in chronic wounds. Additionally, there were no significant differences in complications, including pain scores and the incidence of tissue infection, between the two treatment groups.

## Funding

No funding was received for this study.

## Ethical approval

The study protocol was registered in the Thai Clinical Trials Registry (TCTR20240411002). All participants provided written informed consent for the publication of the photographs.

## Author contributions

The authors indicated in parentheses made substantial contributions to the following tasks of research: initial conception (C.P), design (C.P), provision of resources (C.P, T.R,P.Y,A.S,A.L,T,M), collection of data (C.P, T.R), analysis and interpretation (C.P, T.R), writing and revision of paper (C.P) [Chatchai Prulsapong: C.P, Thanaporn Riansrithongkham; T.R, Pangrum Yongchareon; P.Y, Ajana Sivadechathep; A.S, Anucha Likitvong; A.L, Teerasak Mahamongkol; T.M]

## Declaration of competing interest

All authors have no conflicts of interest to declare.
